# Skin-Derived C-Terminal Filaggrin-2 Fragments Are *Pseudomonas aeruginosa*-Directed Antimicrobials Targeting Bacterial Replication

**DOI:** 10.1371/journal.ppat.1005159

**Published:** 2015-09-15

**Authors:** Britta Hansmann, Jens-Michael Schröder, Ulrich Gerstel

**Affiliations:** Department of Dermatology, University Hospital Schleswig-Holstein, Kiel, Germany; University of São Paulo, BRAZIL

## Abstract

Soil- and waterborne bacteria such as *Pseudomonas aeruginosa* are constantly challenging body surfaces. Since infections of healthy skin are unexpectedly rare, we hypothesized that the outermost epidermis, the *stratum corneum*, and sweat glands directly control the growth of *P*. *aeruginosa* by surface-provided antimicrobials. Due to its high abundance in the upper epidermis and eccrine sweat glands, filaggrin-2 (FLG2), a water-insoluble 248 kDa S100 fused-type protein, might possess these innate effector functions. Indeed, recombinant FLG2 C-terminal protein fragments display potent antimicrobial activity against *P*. *aeruginosa* and other Pseudomonads. Moreover, upon cultivation on *stratum corneum*, *P*. *aeruginosa* release FLG2 C-terminus-containing FLG2 fragments from insoluble material, indicating liberation of antimicrobially active FLG2 fragments by the bacteria themselves. Analyses of the underlying antimicrobial mechanism reveal that FLG2 C-terminal fragments do not induce pore formation, as known for many other antimicrobial peptides, but membrane blebbing, suggesting an alternative mode of action. The association of the FLG2 fragment with the inner membrane of treated bacteria and its DNA-binding implicated an interference with the bacterial replication that was confirmed by *in vitro* and *in vivo* replication assays. Probably through *in situ*-activation by soil- and waterborne bacteria such as Pseudomonads, FLG2 interferes with the bacterial replication, terminates their growth on skin surface and thus may contributes to the skin’s antimicrobial defense shield. The apparent absence of FLG2 at certain body surfaces, as in the lung or of burned skin, would explain their higher susceptibility towards *Pseudomonas* infections and make FLG2 C-terminal fragments and their derivatives candidates for new *Pseudomonas*-targeting antimicrobials.

## Author Summary

*Pseudomonas aeruginosa* is able to cause severe infections that increasingly threaten patients with cystic fibrosis and burns. The emerging antibiotic resistance of those bacteria exigently necessitates the development of new effective drugs. Since healthy skin is unexpectedly resistant towards *P*. *aeruginosa* infections, it constitutes a promising source of new antimicrobials. We identified fragments of the insoluble skin protein filaggrin-2 as *P*. *aeruginosa*-bactericidal proteins that can be released by the action of *P*. *aeruginosa* from the outermost skin layer. Unlike many other antimicrobial proteins, filaggrin-2 fragments target bacterial replication, thus presenting a new mode of antibacterial action. Our findings could initiate the urgent development of newly designed antimicrobials and effectively tackle the challenges of *Pseudomonas* infections

## Introduction

Human skin and mucosal surfaces harbor and encounter a high number of diverse microorganisms [1,2], but are rarely infected. Apart from the physical skin barrier of the *stratum corneum* (reviewed in ref.[3]), a “chemical barrier”, consisting of various antimicrobial peptides and proteins (AMPs), was described to act as innate defense barrier and contributes to the control of microbial growth at body surfaces [4,5]. AMPs can be expressed constitutively or induced by microbe-associated molecular patterns—even at a distance across the physical barrier [6]. In recent investigations a significant role was also ascribed to AMPs as coordinators or alarmins of the adaptive immune response [7]. Despite the increasing number of identified AMPs, there is a growing number of reports describing bacterial defense and immune escape mechanisms to these proteins [8–11] implying that epithelial layers possess additional defense strategies.

Humans are always in contact with microbes, in particular with those from aquatic environment and soil. Especially ubiquitous Pseudomonads are highly abundant on human skin [12] but cause skin infections only when the cutaneous barrier is disturbed, such as in toe web infections [13] or hot tub folliculitis [14]. More severely, under conditions where the cutaneous barrier is completely missing as in burn wounds, *Pseudomonas aeruginosa* is a major cause of morbidity and mortality [15].

As waterborne bacteria, Pseudomonads would thrive at healthy human skin surfaces primarily on areas with an adequate content of moisture and humidity. Besides mucosal epithelia, suitable areas seem to be the lumen and ducts of eccrine sweat glands of skin. Eccrine sweat glands are producing the sweat-specific antimicrobial peptide dermcidin [16]. Interestingly, whereas dermcidin shows antimicrobial activity against various bacteria, there is hardly any against *P*. *aeruginosa* [17]. This surprising finding suggests that sweat glands and the epidermis are producing additional, yet unknown factors, which control the growth of especially *Pseudomonas spp*..

The sole of the foot is rich in sweat glands and extracts of plantar *stratum corneum* have been shown to be a rich source of antimicrobial peptides [18–21]. Heparin-bound compounds of these extracts were subjected to high performance liquid chromatography and peptide fragments of filaggrin-2 (FLG2), formerly also known as ifapsoriasin [22,23], were identified by MS/MS analyses in antimicrobially active fractions.

To test our hypothesis if protein fragments of FLG2 contribute to the resistance of human skin against *P*. *aeruginosa* infections, recombinant FLG2 protein fragments of the 248 kDa full length protein, representing typical regions of the protein, were generated and their antimicrobial properties investigated. Our results show that C-terminal FLG2 protein fragments are able to kill Gram-negative bacteria, and here most efficiently *P*. *aeruginosa*, by interfering with the bacterial replication.

## Results

### FLG2 immunoreactivity in sweat glands and sweat

**Fig 1:**
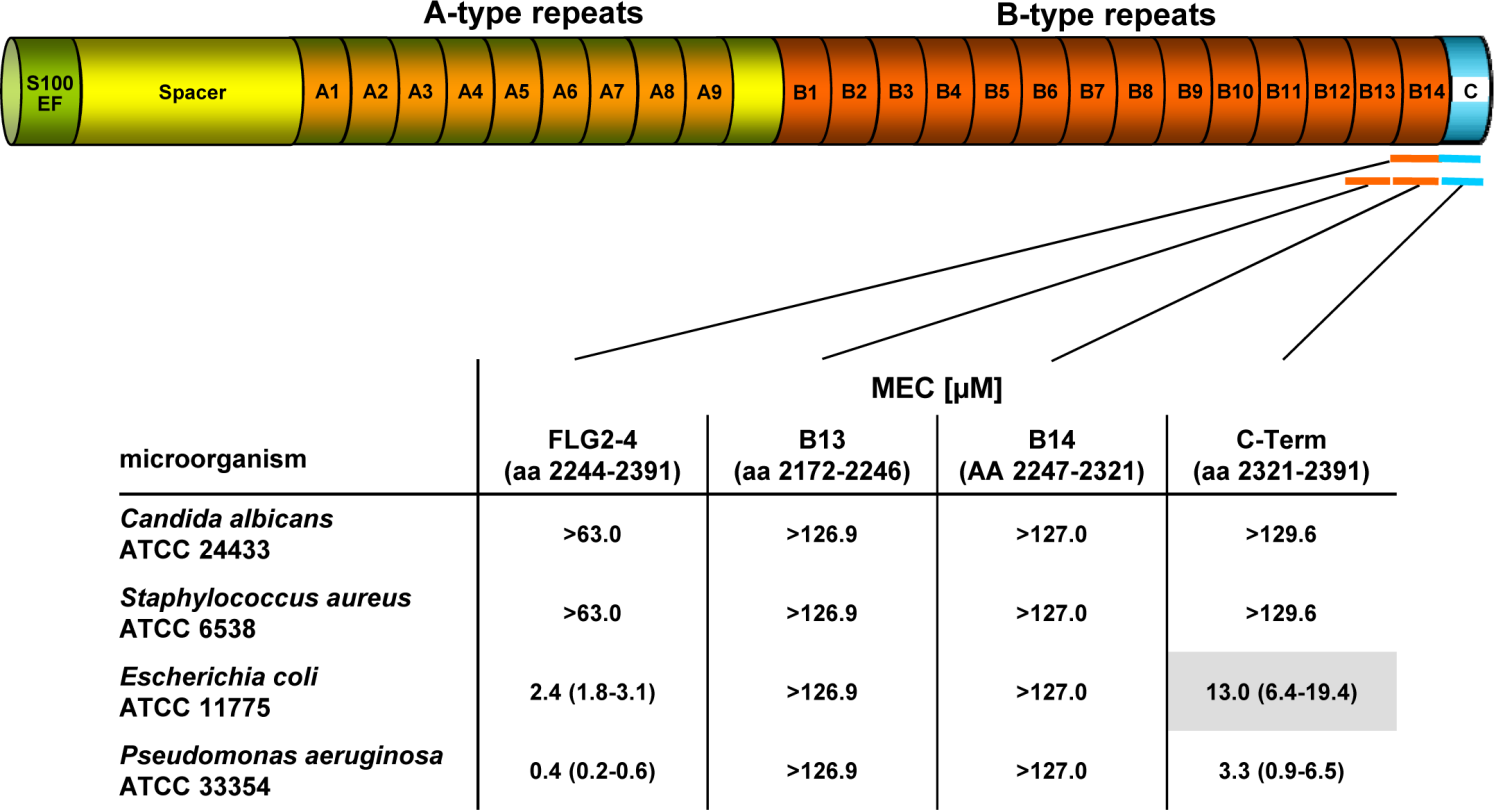
Antimicrobial activity of FLG2 C-terminal fragments tested against various bacteria in the radial diffusion assay. The corresponding amino acids of the full length protein are indicated below each fragment. The MEC is defined as the “minimal effective concentration” by the x-axis intersect in graphical analyses of clearing zones. The MECs stated represent the x-axis intersect of a regression calculated over all measured clearing zone units, the 95% confidence interval is indicated in parentheses; n = 3–12. Shaded gray: turbid clearing zones.

Immunodetection revealed the presence of FLG2 in *stratum granulosum* and *stratum corneum* [23–26] (S1 Fig), and FLG2 fragments were also detected in *stratum corneum* extracts [23]. To test the hypothesis that, apart from its physical barrier function, FLG2 contributes to the innate skin defense, the following FLG2 protein fragments were recombinantly generated: a protein fragment consisting of both the last repeat domain B14 together with the C-terminus (FLG2-4, amino acids 2244–2391), the FLG2 B-repeats B13 (FLG2-B13, aa 2172–2246) and B14 (FLG2-B14, aa 2247–2321), as well as the FLG2 C-terminus (FLG2-C-Term, aa 2322–2391) (Fig 1). In addition, corresponding polyclonal antibodies were generated by immunizing goats with recombinant FLG2 fragments and purified by protein G- and affinity chromatography.

**Fig 2:**
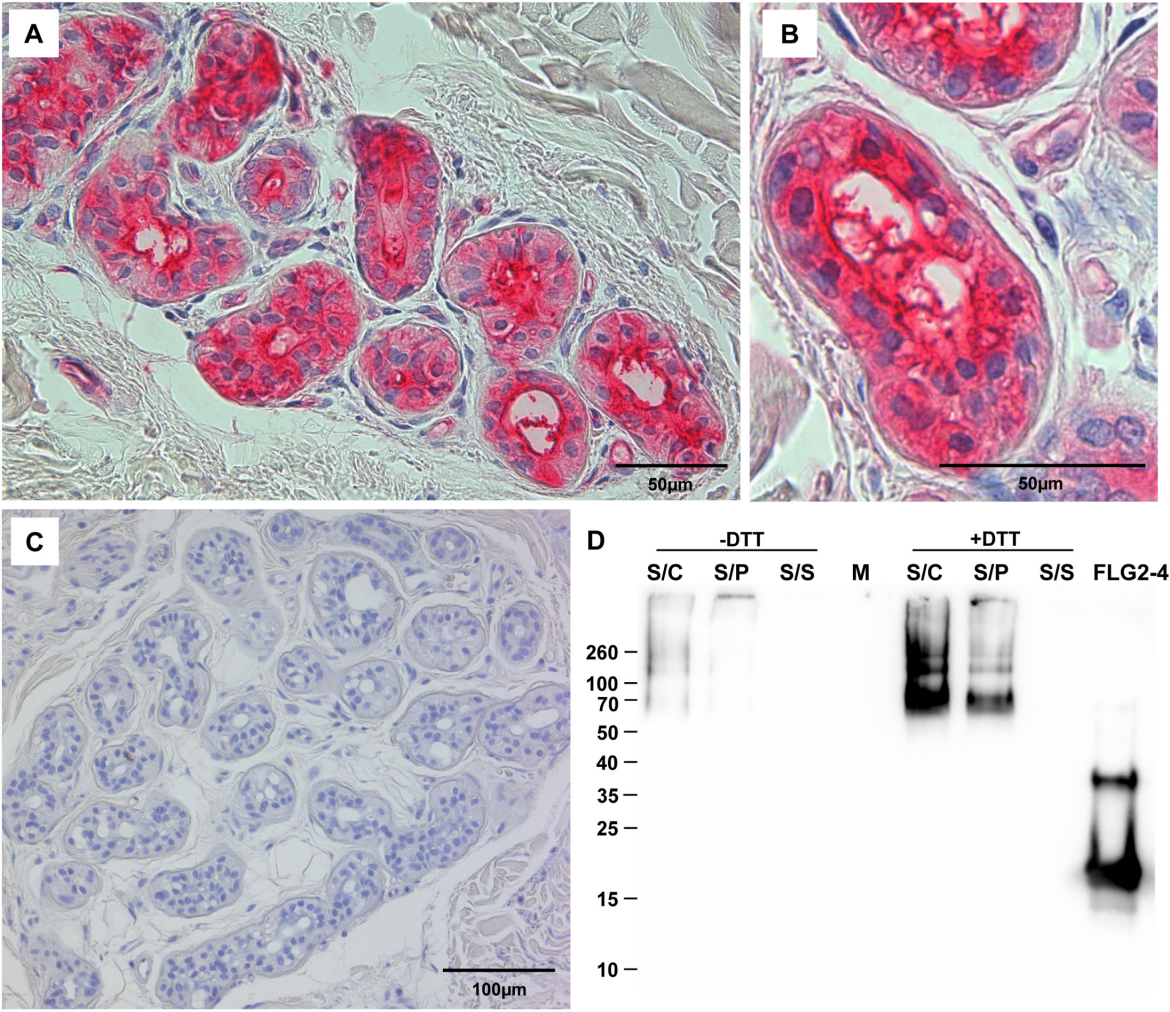
FLG2 is detected in eccrine sweat glands and sweat. A-C: Immunohistochemistry in skin sections. Especially eccrine sweat glands were stained using the antibody directed against FLG2-4 (A, B). C: control section without primary antibody. D: Western Blot analysis of sweat shows an intense staining for FLG2-4 above 70 kDa only in samples pretreated for 1h with 10 mM DTT. A representative experiment out of four is shown. S/C: complete sweat sample, S/P: sweat pellet resuspended in PBS, S/S: sweat supernatant, separated by centrifugation. FLG2: FLG2-4 recombinant fragment. Approximate molecular mass in kDa is indicated on the left.

Immunohistochemistry revealed strong FLG2 immunostaining in the thickened *stratum corneum* of palmar (S1 Fig and [23]) and plantar skin [23], areas that are frequently in contact with the environment and particularly rich in eccrine sweat glands. To investigate whether FLG2 protein is also present in eccrine sweat glands, plantar biopsy material was analyzed with antibodies directed against C-terminal FLG2-4 and intense immunoreactivity was found in sweat gland cells (Fig 2A and 2B). Interestingly, glands showed also luminal FLG2 immunoreactivity with strong staining associated with the luminal surface of the ducts as well as luminal particle-like structures. To investigate whether FLG2 is also present in sweat, sediments as well as supernatants of collected healthy donors' sweat were separately analyzed by Western blotting (Fig 2D). FLG2-4 staining was almost exclusively detected in DTT-treated sediments with the most intense bands corresponding to 70 and 140 kDa, suggesting that sweat gland FLG2 is crosslinked to particular matter and needs to be processed to release C-terminus-containing FLG2 protein fragments.

### The FLG2 C-terminal protein fragment is antimicrobially active against various *Pseudomonas spp*.

Especially plantar and palmar skin is frequently in contact with soil- and/or waterborne microbes. Hence, it is tempting to speculate that these skin areas are protected from infections by Pseudomonads by means of water-insoluble, *stratum corneum*-associated antimicrobial compounds. To test this hypothesis, the antimicrobial activity of the different recombinantly expressed FLG2 protein fragments was analyzed in the radial diffusion assay (RDA) system and identified the FLG2 C-terminal protein fragment (FLG2-4) as potent *Pseudomonas aeruginosa* ATCC 33354 killing factor with a minimal effective concentration (MEC) of 0.4 μM in this assay system (Fig 1). Whereas FLG2-4 was also bactericidal for *Escherichia coli* ATCC 11775 (MEC: 2.4 μM), no killing activity was seen for the yeast *Candida albicans* ATCC 24433 and the Gram-positive *Staphylococcus aureus* ATCC 6538 (Fig 1). FLG2-4 is composed of the FLG2 repeat domain B14 and the FLG2-C-terminus. In order to identify the structural elements responsible for bactericidal activity within the FLG2-4 fragment, FLG2-B14, the FLG2-C-terminal fragment (C-Term), and the second to last repeat, FLG2-B13, were analyzed in the RDA system. The antimicrobial activity against the tested bacteria was solely located within the FLG2 C-Term protein fragment, although the MECs for *E*. *coli* ATCC 11775 (13.0 μM) and *P*. *aeruginosa* ATCC 33358 (3.3 μM) were about 5.5–8.3 times higher than seen for the FLG2-4 (Fig 1).

With the hypothesis that the C-terminal fragment FLG2-4 is active against various soil- and waterborne bacteria, further *P*. *aeruginosa* strains, including *P*. *aeruginosa* ATCC 10145, 33348, 333358, 39324, PAO1, the Cystic Fibrosis (CF) clinical isolates CF 636, 640, 645, 646, and other *Pseudomonas sp*. as *P*. *stutzeri* RV A2/1990, *P*. *syringae* ATCC 10205, *P*. *paucimobilis* RV A2/1994, *P*. *fluorescens* ATCC 49323, and *P*. *putida* RV A1/2000 were tested. Antimicrobial activity was observed against all tested strains with MECs between 0.1 and 7.0 μM (S1 Table). Most of the cystic fibrosis (CF) clinical isolates as well as the *P*. *aeruginosa* strain ATCC 39324, *P*. *fluorescens* ATCC 49323, and *P*. *putida* RV A1/2000 showed turbidity within the clearing zones. Since all of the subsequent experiments were performed in liquid systems, we also analyzed antimicrobial activity of FLG2-4 against *E*. *coli* ATCC 11775 and *P*. *aeruginosa* ATCC 33354 in the microdilution assay system; the LD_90_ of 0.8 μM and 0.2 μM, respectively, confirmed bactericidal activity found in the RDA (S2 Table).

In summary, these results indicate that the C-terminal peptide fragment of FLG2, FLG2-4, is a potent, preferentially *Pseudomonas ssp*. killing antimicrobial peptide. Whereas the antimicrobial activity is located solely within the C-terminus, the presence of the last B-repeat (B14) increases the killing potency of the protein.

### FLG2 release and uptake by *P*. *aeruginosa*

**Fig 3:**
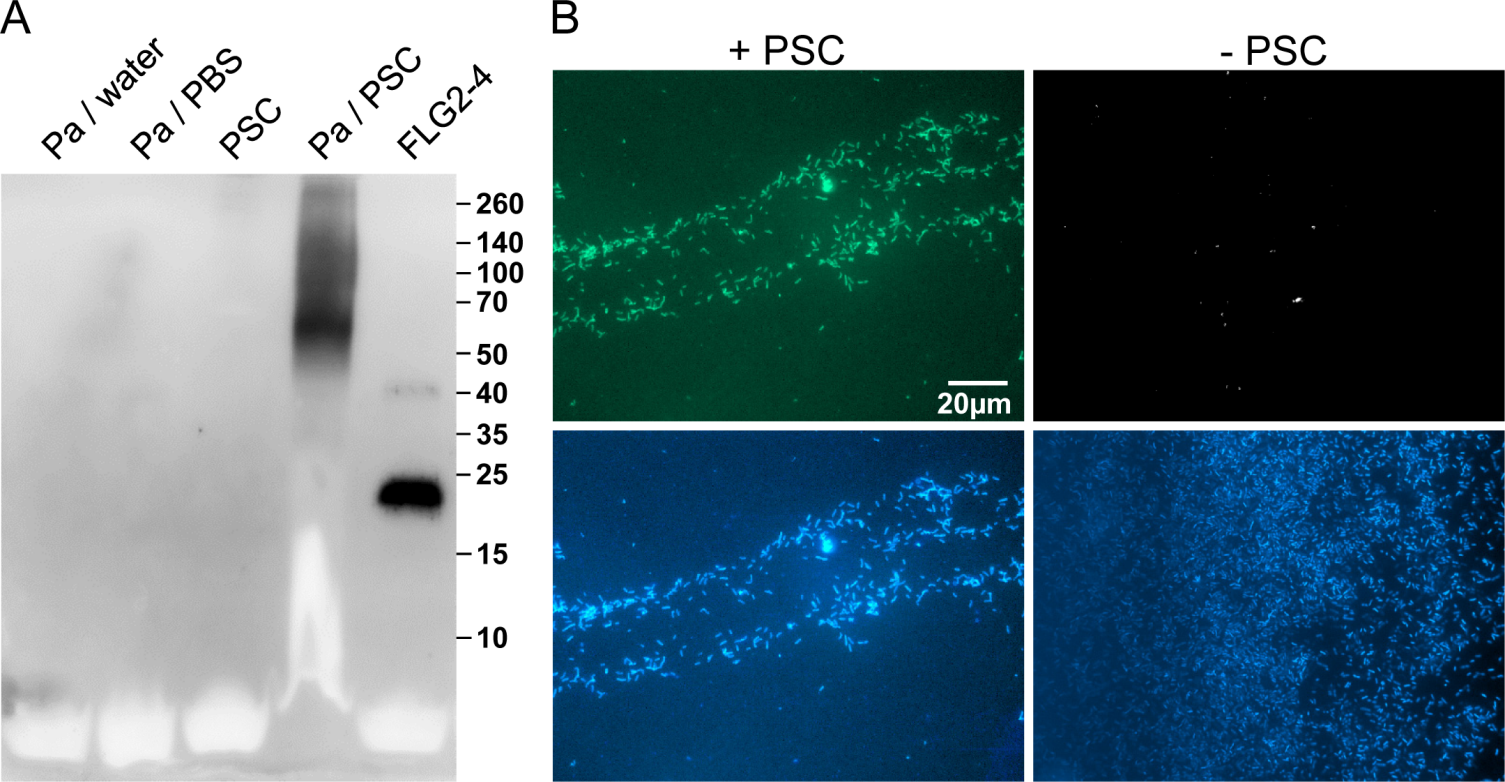
*P*. *aeruginosa* released and accumulated FLG2-4 immunoreactivity from plantar stratum corneum. A: *P*. *aeruginosa* (Pa) ATCC 33354 were grown in presence or absence of plantar stratum corneum (PSC) and supernatants were subjected to Western Blot analyses using anti-FLG2-4 antibodies. Supernatants of P. aeruginosa grown in water or PBS and an untreated PSC supernatant served as control. Approximate molecular mass in kDa is indicated on the right. B: Immunofluorescence microscopy of P. aeruginosa ATCC 33354 co-incubated with (left panel) or without PSC (right panel) using anti-FLG2-4 antibodies (top panels) and DAPI nuclear counterstain (bottom panels).

Immunoreactive FLG2 fragments are present in sweat and extraction was only possible in the presence of the reducing agent DTT (Fig 2D). Also, FLG2 extraction from *stratum corneum* was seen to be possible in the presence of SDS and DTT [23], non-ionic detergents, 8M urea or EDTA [24]. The observation of smaller immunostained bands, apart from a band of the expected size of the full length protein, suggests that FLG2 is cleaved within the transition zone from *stratum granulosum* to *stratum corneum* and sweat glands, presumably necessary for generation of soluble antimicrobially active fragments. *P*. *aeruginosa* is metabolically versatile [27] and known for the degradation of even insoluble substances [28]. Therefore, *P*. *aeruginosa* was cultivated with plantar *stratum corneum* (PSC) and the release of immunoreactive C-terminal FLG2-fragments was monitored. In this experimental system, *P*. *aeruginosa* was able to break down FLG2 fragments from *stratum corneum* that were recognized by the specific C-terminal directed FLG2-4 antibody (Fig 3A). Interestingly, the major bands between 50 and 70 kDa seem to resemble that of DTT-treated sweat (compare Figs 3A and 2D).

The efficacy of antimicrobials highly depends on the type of interaction with their target microbes. To analyze whether FLG2 breakdown products of PSC associate with or are taken up by the pathogen, *P*. *aeruginosa* was spatially separated from PSC by a transwell (pore size 0.45 μm). After incubation, bacterial cells were taken from the upper transwell compartment, washed, and subjected to immunofluorescence analyses. Despite the separation of *P*. *aeruginosa* and *stratum corneum*, bacterial cells showed strong FLG2-4 immunoreactivity (Fig 3B, left panel) when compared to untreated cells (Fig 3B, right panel).

### The C-terminal FLG2 protein fragment FLG2-4 causes bleb formation in *P*. *aeruginosa*

**Fig 4:**
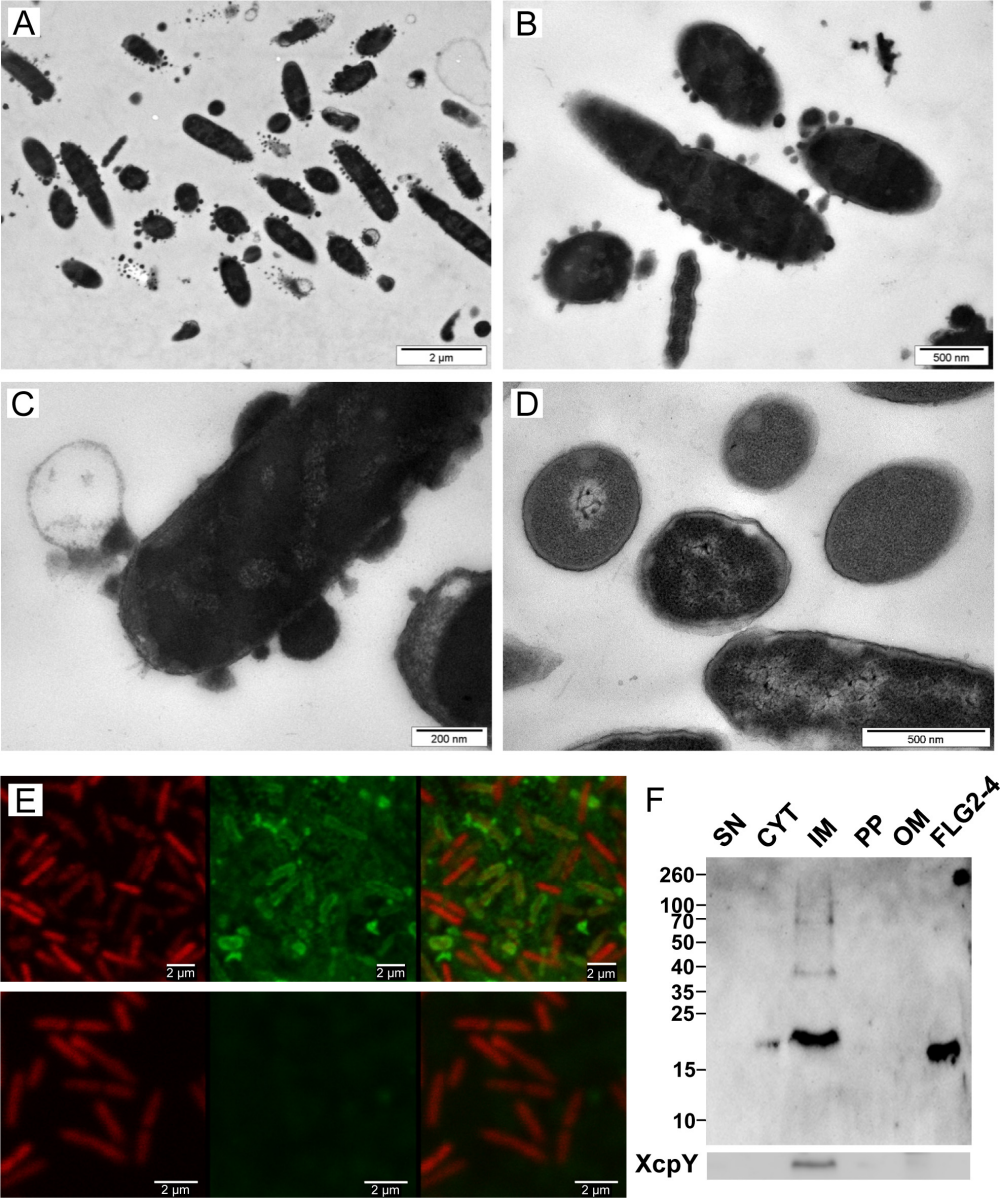
Effect and localization of FLG2-4 on *P*. *aeruginosa*. A-D: Electron microscopy of *P*. *aeruginosa* ATCC 33354 treated with FLG2-4 for 30 min (A-C) and untreated (D); E: Confocal laser scanning microscopy of FLG2-4-treated *P*. *aeruginosa* ATCC 33354, left panels: DRAQ5 staining, middle panels: FLG2-4 staining, left panels: merged images, upper panels: FLG2-4 treated bacteria, lower panels: untreated bacteria; F: Western Blot of fractionated, FLG2-4-treated, *P*. *aeruginosa* PAO1. Beneath, inner membrane fraction, localized by western blotting using antibodies directed against XcpY; SN: sample supernatant, CYT: cytoplasmic, IM: inner membrane, PP: periplasmic, OM: outer membrane enriched fractions, FLG2-4: FLG2-4 recombinant fragment. Approximate molecular mass in kDa is indicated on the left.

To get further insight into the mechanism by which FLG2-4 kills *P*. *aeruginosa*, morphology of FLG2-4-exposed *P*. *aeruginosa* were examined by transmission electron microscopy (TEM). As shown in Fig 4A, 4B and 4C, *P*. *aeruginosa* exhibits massive bleb formation already within 30 min of treatment, when compared to control bacteria (Fig 4D). Prolonging exposure to FLG2-4 resulted eventually in a significant destruction of the bacterial cells (S2 Fig).

To localize FLG2-4 during treatment, confocal laser scanning microscopy was performed. FLG2-4 immunofluorescence could be detected mainly at or within the membrane of the bacteria (Fig 4E) and was consistent with the subcellular fractionation of FLG2-4-treated bacteria (Fig 4F). Nonetheless, confocal laser scanning microscopy revealed also FLG2-4 immunoreactivity within the blebs released *by P*. *aeruginosa* (S3 Fig).

### Antimicrobial mode of action of FLG2-4

**Fig 5:**
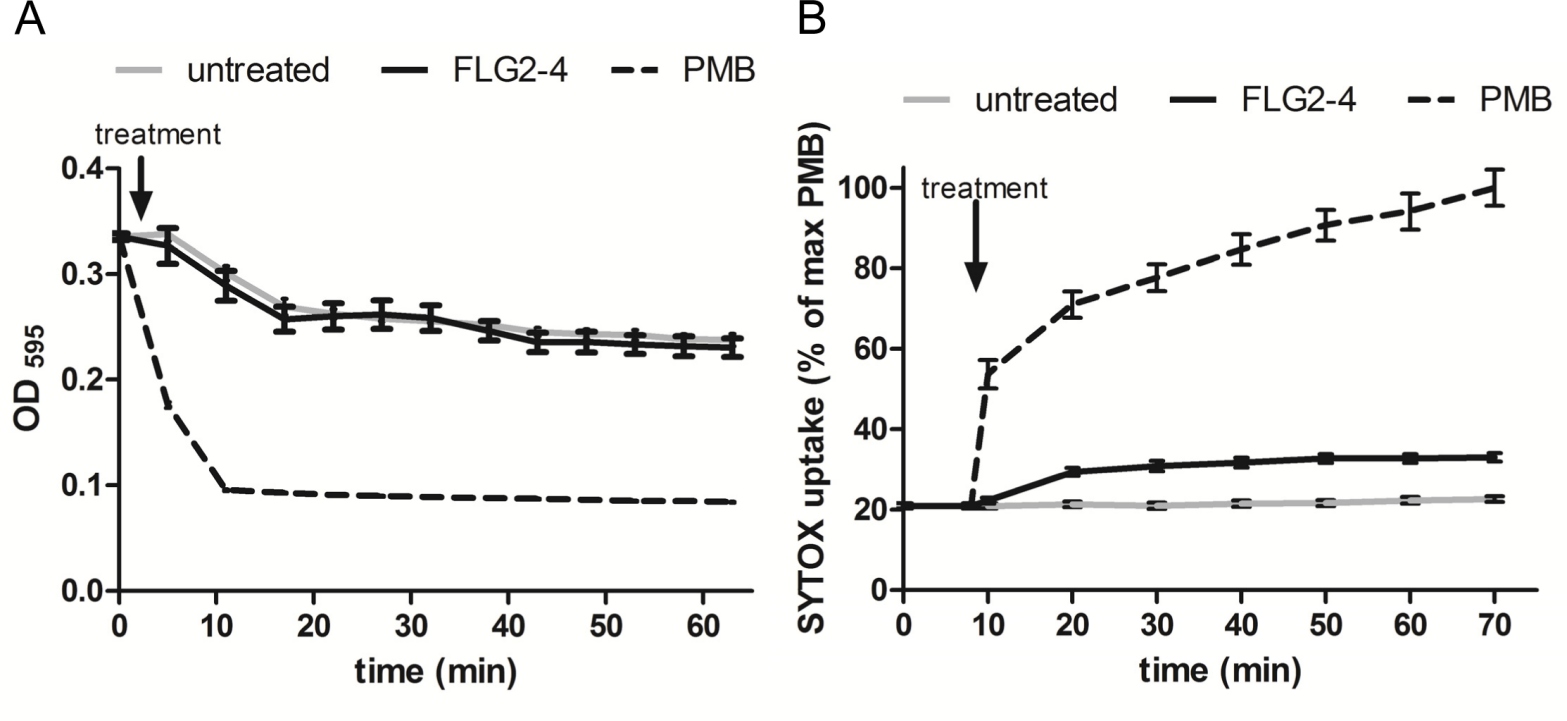
FLG2-4 does not permeabilize the bacterial membrane. A: Lysis by 50 μg/ml lysozyme (expressed as OD_595nm_ against time) of chloramphenicol-treated *P*. *aeruginosa* PAO1 cells (gray line) in presence of Polymyxin B (PMB, 7.7 μM; dotted line) or FLG2-4 (0.8 μM; black line). B: SYTOX Green uptake by *P*. *aeruginosa* PAO1 treated with PMB (2 μM; dotted line), FLG2-4 (0.8μM; black line) or kept untreated (gray line). Results are expressed as percentage of maximum SYTOX Green uptake in the presence of PMB. Data represent means of three experiments ± SD.

Bleb formation of *P*. *aeruginosa* provides an active external decoy to trap cell surface-acting antimicrobial agents [29], and is described as a sign of perturbations in the bacterial envelope [30] or DNA damage [31]. Analyzing the effect of FLG2-4 treatment on the bacterial outer membrane, a lysozyme lysis assay was performed in the presence of chloramphenicol to prevent self-induced lysis of *P*. *aeruginosa*. As shown in Fig 5A, FLG2-4 treatment did not impair the integrity of the outer membrane by means of lysozyme penetration. FLG2-4-treated bacteria do not exhibit a difference in the OD_595nm_ compared to control cells. Using the pore forming antibiotic Polymyxin B [32,33], treated bacteria showed a marked decrease in OD already within less than 10 min. Furthermore, the uptake of the membrane-impermeable DNA-stain SYTOX Green was only slightly increased during the first 10 min after FLG2-4 treatment (Fig 5B), while Polymyxin B treatment showed an increased uptake of SYTOX Green almost immediately after addition to the bacterial cells.

### FLG2-4 interacts with DNA

**Fig 6:**
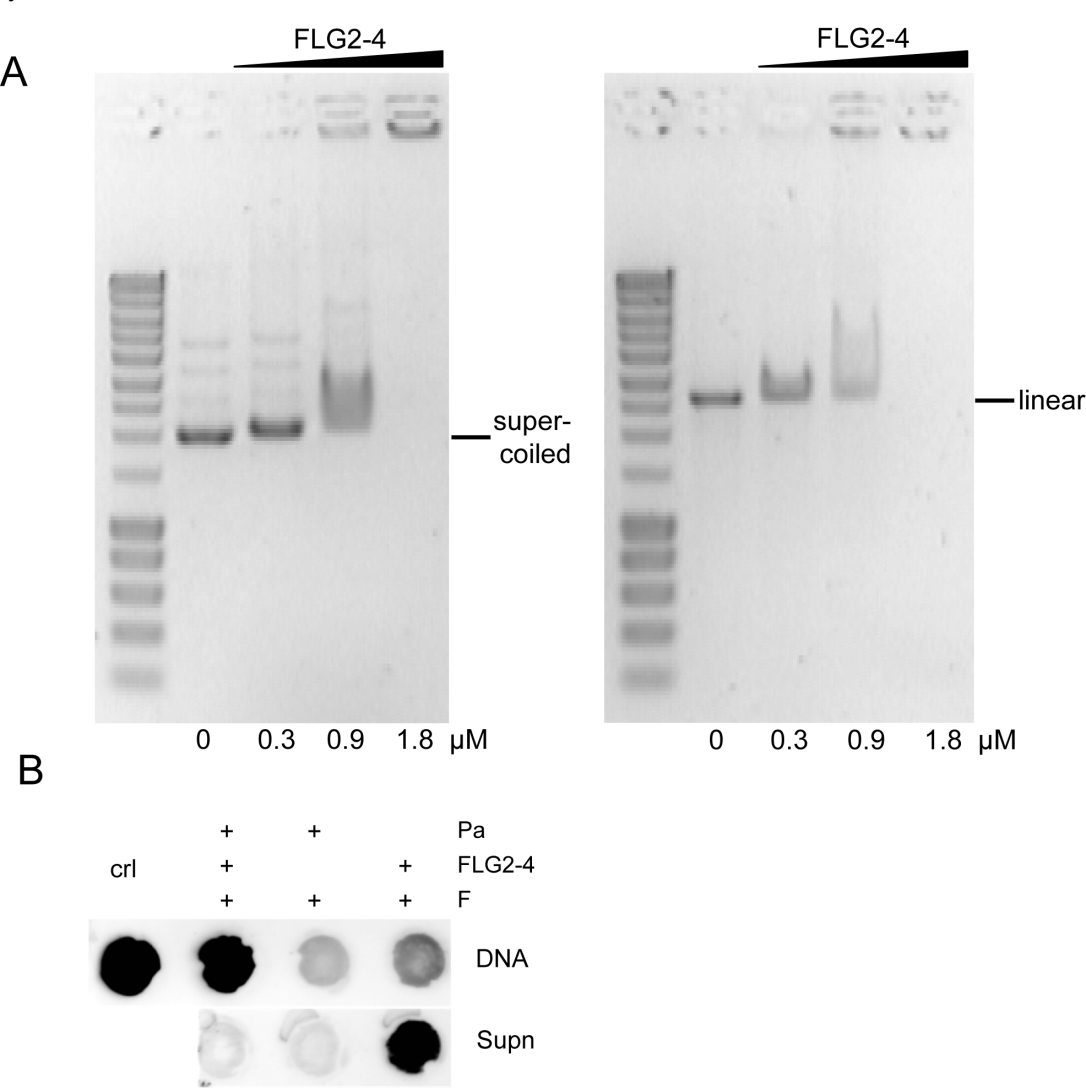
Interaction of FLG2-4 with DNA. A: Electrophoretic shift assays of ~120 ng supercoiled (left panel) and linearized (right panel) plasmid DNA with increasing concentrations of the FLG2-4 fragment. Used concentrations are indicated below the lanes. B: *In vivo* crosslinking of FLG2-4 to DNA. *P*. *aeruginosa* (Pa) were treated with FLG2-4 or left untreated, subsequently FLG2-4 DNA interactions were crosslinked with formaldehyde (F) and isolated DNA and non-precipitated fraction (Supn) were monitored for the presence of FLG2-4 by dot-blot analyses. Crl: FLG2-4.

Since the observed blebbing might also be a reaction to DNA damage (31), the ability of FLG2-4 to interact with DNA was analyzed. Electromobility shift assays showed that the basic protein FLG2-4 (pI: 10.18) is able to shift supercoiled as well as linearized plasmid DNA, when using concentrations in the range of the LD_90_ for the tested Gram-negative bacteria (Fig 6A). Interestingly, the antimicrobially active C-terminal peptide (pI: 9.8) also interacted with and shifted DNA (S4 Fig), but even though having almost identical pI values, neither the antimicrobially inactive FLG2-B13 (pI: 9.95) nor FLG2-B14 peptide (pI: 8.59) caused a DNA shift (S4 Fig). To prove an *in vivo* interaction of FLG2-4 with the bacterial DNA, treated *P*. *aeruginosa* were fixed with formaldehyde and subsequently DNA was isolated. FLG2-4 crosslinking to DNA was monitored by dot-blot analyses using the anti FLG2-4 antibody and reveal that FLG2-4 was detected within the DNA fraction (Fig 6B).

### FLG2-4 interferes with the polymerase chain reaction

**Fig 7:**
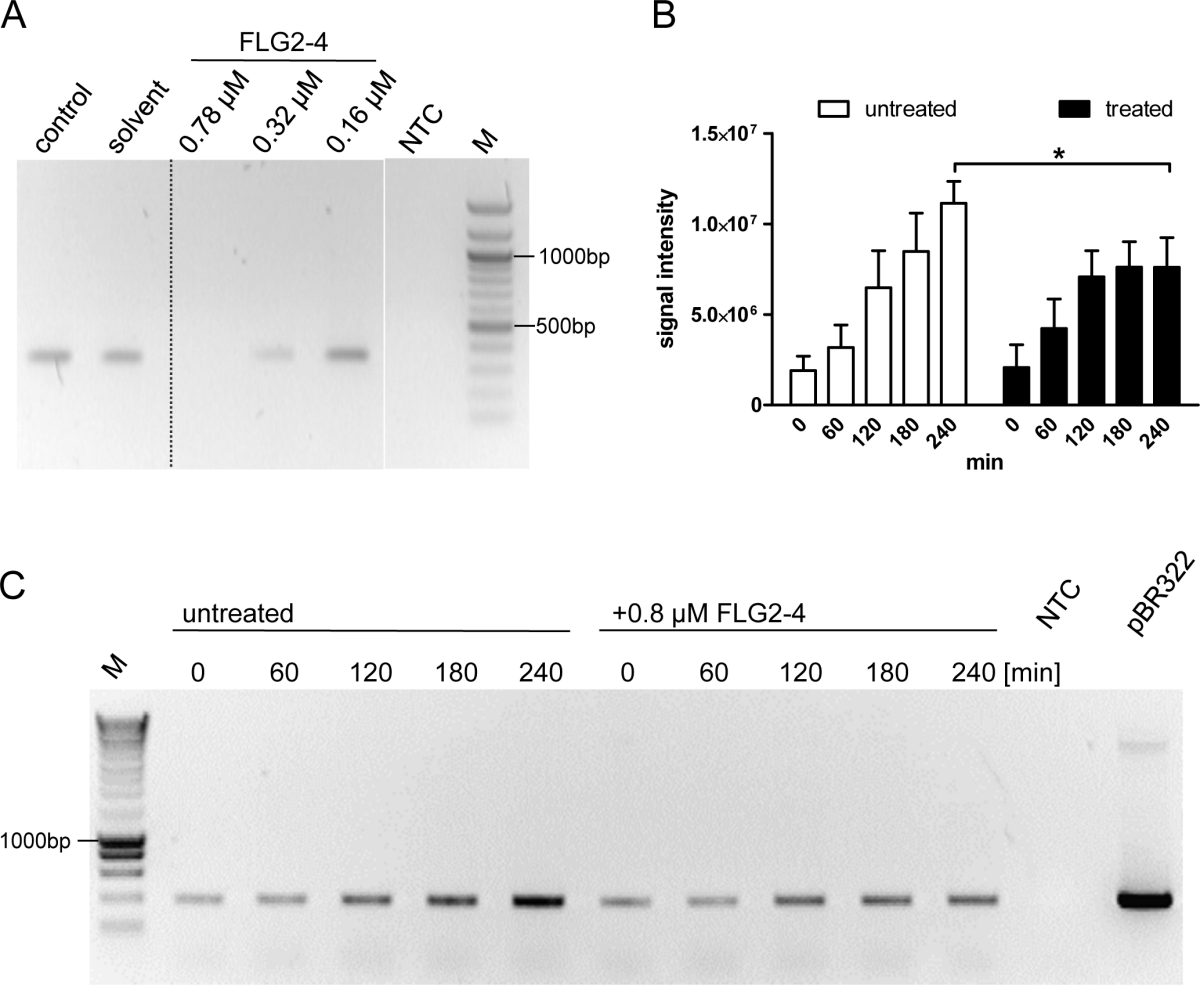
FLG2-4 is able to inhibit bacterial replication. A: PCR *in vitro* inhibition assay. Different concentrations of FLG2-4 were tested for PCR-inhibiting properties. A GAPDH fragment was amplified from human keratinocyte cDNA. Control: PCR reaction without FLG2-4, solvent: solvent of FLG2-4 (0.01% acetic acid/0.02% NaN_3_). A representative experiment out of twelve is shown. B: Densitometric analyses of semiquantitative PCRs of *in vivo* replication assays. Amplicons separated by agarose gels were stained with ethidium bromide and subjected to densitometric analysis. The error bars represents SD, significance was calculated using a two-way ANOVA and a Bonferroni post hoc test, n = 4. C: Exemplary *in vivo* replication inhibition PCR over indicated time periods. NTC: no template control, M: molecular mass marker,*: p<0.05, pBR322: positive control using 5ng pBR322 directly as template.

Many cationic proteins display binding affinities to DNA, e.g. LL37 [34], thereby affecting also replication events [35]. Therefore, the impact of FLG2-4 was investigated in a simplified model of bacterial DNA replication, the polymerase chain reaction (PCR). Using conventional *Taq* Polymerase, PCR analyses were performed in the absence and presence of FLG2-4. Whereas concentrations of 0.32 μM and 0.16 μM did not affect the reactions, in samples containing FLG2-4 at concentrations reflecting the LD_90_ for *E*. *coli* (0.78 μM or 250 ng/20μl), no amplicon was detected using GAPDH primers on cDNA (Fig 7A). Even coincubation of the FLG2-B13 and FLG2-B14 protein fragments did not hamper the FLG2-4 mediated PCR inhibition (S5A Fig). Merely increasing the polymerase concentration in the PCR reactions could partly abolish the FLG2-4 inhibition (S5C Fig). Neither the structurally unrelated AMP hBD-2 (corresponds to ~1.25 fold of LD_90_ for *E*. *coli* [36]) nor BSA did affect PCR reactions at the applied concentrations (S5B Fig).

### FLG2-4 affects bacterial replication *in vivo*

To test the effect of FLG2-4 on bacterial replication *in vivo*, a plasmid-based replication assay was developed. The assay based on the finding that following chloramphenicol treatment, the FLG2-4-induced lysis of bacteria is similarly prevented (Fig 5A) as known for some quinolone antibiotics [37,38]. Since in the presence of the translation-inhibitor chloramphenicol bacterial chromosomal replication is also blocked [39] (S5D Fig), plasmid replication of pBR322 was monitored in *E*. *coli* ATCC 11775 over time. This plasmid, which contains a closely related origin of replication (*ori*) of the ColE1, the pMB1 *ori*, replicates in an autonomous, self-controlled way [40]. While plasmid concentration increased over time in bacteria treated solely with chloramphenicol, addition of FLG2-4 in concentrations reflecting its LD_90_ (0.8 μM) impede plasmid replication (Fig 7B and 7C).

## Discussion

Human skin acts as barrier between the outer environment and the susceptible body interior. To manage this task, the cells of the epidermis, the keratinocytes, undergo a complex and tightly regulated process called epidermal terminal differentiation. Eventually this process causes the formation of a protective physical skin barrier, the *stratum corneum*. This cell layer, consisting of dead cornified keratinocytes, called corneocytes, and lipids, blankets the vulnerable layers of living keratinocytes. The keratinocytes themselves are able to induce more complex innate immune responses, e.g. by providing several constitutive and inducible antimicrobial peptides to protect skin from invading microbes [5,41,42]. Eccrine sweat glands support the maintenance of the barrier by secreting antimicrobial fragments of dermcidin [43] or supporting the process of re-epithelialization during wound healing [44].

Upon endo- or exogenous breakdown of *stratum corneum* compounds, C-terminus-containing FLG2 protein fragments are generated beyond the living epidermis. Here we could show that C-terminal FLG2 fragments exhibit antimicrobial activity, preferentially against *P*. *aeruginosa* and other water or soil bacteria, which are constantly challenging our skin. In contrast to the mechanisms assigned to many other AMPs [45–48], the bactericidal mechanism of FLG2-4 is not based on pore formation by inserting into membranes. Instead, our results show that association of FLG2-4 with the cytoplasmic membrane and impediment of the bacterial replication machinery causes the bacterial death. Similar to the quinolone antibiotics that interfere with the bacterial replication by acting on its DNA gyrase finally causing the bacterial death [49,50], our results suggest that FLG2-4 impair the DNA polymerases processivity, thereby stalling bacterial replication and thus preventing the survival of certain pathogens on or in human skin.

As the majority of the proteins involved in terminal differentiation and formation of the cornified cell envelope, the S100 fused-type protein (SFTP) FLG2 is encoded within the epidermal differentiation complex on chromosome 1q21.3 [51]. In human epidermis, FLG2 colocalizes partially with filaggrin, one of the best characterized member of the SFTP family [23,24,26]. Similar to filaggrin, or its precursor profilaggrin, FLG2 is proteolytically processed during epidermal terminal differentiation [23] and possesses similar biochemical properties [24]. The biological functions of FLG2 are mostly unknown. The N-terminal part of FLG2, including the A-type repeats, shares identity to the SFTP member hornerin, whereas the C-terminal part, including the B-repeats, rather shows similarities to filaggrin [23]. For the repetitive region of the murine B-repeats a keratin-bundling activity similar to filaggrin subunits has been shown [52], assuming a similar function for the human FLG2 B-repeats. The antimicrobially active C-terminal region, however, does not belong to the repetitive sequences. About the function of the FLG2 C-terminus just as little is known as for the filaggrin C-terminus; it was suggested that it is necessary for proper processing of the precursor profilaggrin into smaller subunits [53,54]. Maybe an equally important role, in addition to the antimicrobial properties, can be ascribed to the C-terminus of FLG2.

As potential crosslinking partner of the cornified cell envelope, FLG2 seems to be retained within the *stratum corneum* (S1 Fig) and is somehow particle-bound and hardly water-soluble in eccrine sweat (Fig 2). FLG2 is detectable in the *stratum corneum*, especially of palmar and plantar skin [23]. These skin locations, enriched in eccrine sweat glands, in principle provide proper habitats for soil- and waterborne pathogens as *Pseudomonas spp*.. Even though our hands and feet are frequently in contact with the outer environment and therefore with a multiplicity of these microbes, infections are virtually excluded.

The adaptable, non-stringent metabolic requirements enable *Pseudomonas spp*. to occupy a diversity of biological niches. Regarding its keratinolytic [55] and ceramidase activity [56] as well as its frequent detection on human skin [12], it is not astonishing that *P*. *aeruginosa* is able to break down C-terminus-containing FLG2 fragments from *stratum corneum* (Fig 3A). More strikingly, these fragments seem to accumulate in bacterial cells (Figs 3B, 4E and 4F). Many antimicrobial peptides interact with the bacterial membrane, leading to pore formation and finally leakage of the intracellular components [57]. Ultrastructural analyses as well as confocal microscopy revealed bleb formation on the bacterial surface shortly after treatment with the C-terminal FLG2-4 fragment. Further, FLG2-4 immunoreactivity could be detected at these blebs and surrounding the cells (S3 Fig, Fig 4E). Due to the high energy consumption, bleb formation of bacteria is a short term bacterial stress response to haul out detrimental factors and thus diminishing the efficacy of AMPs [58]. Especially antimicrobial factors that cause perturbations in the bacterial cell envelope or induce DNA damage, are known to trigger this stress response [29,59]. Subfractionation of FLG2-4-treated *P*. *aeruginosa* particularly showed FLG2-4 immunoreactivity in the inner membrane fraction (Fig 4F). In addition, FLG2-4 interacts with bacterial DNA (Fig 6). These results suggest that FLG2-4 might colocalize and interfere with the membrane-associated bacterial replication complex. The detailed molecular mechanisms of bacterial replication are not fully understood, but replication always starts at one particular site, the origin of replication or *ori*C, and it is a multiprotein process [60]. During the initiation of replication in bacteria, the membrane-associated protein DnaA binds the *ori*C and subsequently enforces unwinding and opening of the double helix, finally enabling the assembly of the replisome [61–64]. In further steps, helicases and topoisomerases unwind and relax the coiled DNA helix. Once the DNA is melted and primed, the general DNA replication process starts. The presence of FLG2-4 in the inner membrane fractions and its ability to bind DNA indicated an interference with the DNA polymerase reaction, the central stage of replication. In concordance, in a first, simplified model of replication, FLG2-4 was able to stall the *in vitro* polymerase chain reaction in a dose-dependent manner (Fig 7A). This effect could be rescued partly by increasing the polymerase concentration (S5C Fig). To monitor the effect of FLG2-4 on *in vivo* replication, an assay was developed that avoided the stress-induced disintegration of the bacterial cell shortly after FLG2-4 treatment by addition of the translation inhibitor chloramphenicol. Since chloramphenicol also blocks the replication of the bacterial chromosome [65], the autonomous replication of the transformed pBR322 plasmid in the presence of chloramphenicol [39,66] was investigated and, in support of our hypothesis, it was found to be significantly reduced upon FLG2-4 treatment in *E*. *coli* (Fig 7B and 7C).

Taken together, these results lead to a model where *Pseudomonas spp*., when encountering human skin, are capable of degrading available substrates (e.g. FLG2 among other proteins of the *stratum corneum*), and in turn generate C-terminus-containing FLG2 fragments. These fragments accumulate at the bacterial surface, eventually integrate into the bacterial inner membrane and interfere with the replication machinery. In consequence, the inhibition of the replication induces a bacterial SOS response, similar as observed for certain quinolone antibiotics [37,38] that ultimately causes bacterial cell death [49,50].

The present study indicates that C-terminal FLG2 fragments might be promising alternatives to traditional antibiotics used to cure especially *Pseudomonas* infections. Considering the fact that FLG2 transcripts could not been detected in the human lung [23] (nor its mouse orthologue in the murine lung [67]) might emphasize a potential application of C-terminal FLG2 fragments in the eradication of early *P*. *aeruginosa* infection in cystic fibrosis in order to prevent a chronic infection state and its clinical consequences.

## Material and Methods

### Expression of FLG2-4 and antibody generation

The expression and protein synthesis was performed as described before [23]. The protein fragment for the C-terminal fragment of FLG2, FLG2-4, was produced using the pETSUMO expression system (Invitrogen), whereas the other C-terminal fragments, FLG2-B13,-B14, and-C-Term were produced using the pSUMO3 expression system (LifeSensors). Immobilized metal ion affinity chromatography-purified SUMO-FLG2 fusion proteins were cleaved using SUMO protease 1 and 2 (LifeSensors), respectively, and the FLG2 proteins were subsequently purified by reversed phase HPLC. Polyclonal goat-anti-FLG2-4 antibodies were generated and purified by affinity chromatography with the respective protein fragment, as described earlier [23].

### Ethics statement

All experiments were performed according to the Declaration of Helsinki protocols and under protocols approved by the Ethics Committee at the Medical Faculty of the Christian-Albrechts-University, Kiel (AZ 104/06). Sweat and stratum corneum samples derived from healthy volunteers, skin explants for histology derived from patients undergoing surgery, and were obtained after written informed consent. Commercial production of customized polyclonal antibodies in the goat was in accordance with the German Animal Welfare Act (§10a). The protocol was approved by the ethics committee of the “Landesamt für Landwirtschaft, Lebensmittelsicherheit und Fischerei Mecklenburg-Vorpommern“, Germany (AZ: LVL M-V 7221.3–2.5-010/2004).

### Immunohistochemistry

FLG2 protein localization was visualized in skin paraffin sections as described previously [23].

### Microbial growth conditions

Microbes were cultivated in either brain heart infusion medium (BHI), lysogeny broth (LB), tryptic soy broth (TSB) or M9 medium [68] supplemented with 0.5% casamino acids and 1μg/ml thymine. If not otherwise stated, microbes were incubated under shaking conditions (37°C at 170 rpm).

### Antimicrobial activity assays

The purified proteins were tested against different bacteria in a modified radial diffusion (RDA) or microbroth dilution assay [69]. The underlay agarose used for the RDA was inoculated with approximately 10^5^ colony forming unit (CFU) per ml, the protein fragments were applied using a twofold dilution series. Evaluation was performed by calculating the linear regression over each measured clearing zone replicate (GraphPad Prism version 5.04 for Windows). The x-intercept, that represents the "minimal effective concentration" (MEC) according to [69], was calculated as a regression over all measured clearing zone units. The x-axis intercept and its 95% confidence interval are given in μM for each microorganism. For the microbroth dilution assay, bacteria were grown to early log-phase in BHI, washed with 10 mM sodium phosphate/1% TSB, and adjusted to a concentration of 10^4^−10^5^ CFU/ml, followed by an incubation with FLG2-fragments for 2h at 37°C. The samples were plated out on BHI agar in appropriate dilutions and CFU were counted after an overnight incubation at 37°C. The LD_90_ defines the protein concentration killing 90% or more of the bacteria.

### *Stratum corneum* degradation

Pooled powdered *stratum corneum* (SC)(~15 mg) was exposed to UV light for 60 min to minimize microbial load. Thereafter the SC was washed once with sterile H_2_O. Remaining liquid was removed by a centrifugation step (2000 x g, 5 min) and 200 μl of a logarithmic-grown *P*. *aeruginosa* culture (OD_600_ 0.2) were added. Breakdown products were analyzed by Western Blot using goat-anti-FLG2-4 as described [23].

### Immunofluorescence of bacteria

Autoclaved and washed plantar *stratum corneum* (~15 mg) was placed in a 6-well-microplate. 100μl of a logarithmic-grown *P*. *aeruginosa* culture were transferred onto the *stratum corneum*, separated by a transwell (0. 45μm). After incubation for 30 h at 37°C, bacteria were washed and heat-fixed to glass slides. After blocking with Roti-Block (Roth), immunoreactive FLG2 was detected with goat-anti-FLG2-4 antibody (0.5 mg/ml, 1:100) and monitored using an AlexaFluor488-coupled chicken-anti-goat IgG (Invitrogen) secondary antibody. Slides were analyzed using a Zeiss Axioskop.

### Electron microscopy

Logarithmic grown *P*. *aeruginosa* ATCC 33354 were washed and concentrated to an OD_600nm_ of 4 in 10 mM sodium phosphate/1% TSB. The amount of FLG2-4 used was about 2 x 10^7^ molecules per colony forming unit (CFU). Samples were incubated at 37°C for 30 min, 90 min, and 180 min. After incubation, the samples were fixed in 2.5% glutaraldehyde. Afterwards, the pelleted samples were resuspended in 2% Noble Agar, washed several times with PBS pH 7.4, and incubated with 1% osmium tetroxide in PBS for 45 min. Again, this was followed by several washings in PBS. Samples were then dehydrated in an ascending graded ethanol series. For embedding, the EtOH was replaced stepwise by a polyhydroxy-aromatic acrylic resin (LR White), starting at a resin:EtOH ratio of 1:2, followed by 1:1, 2:1, and three times in resin only, each for 30 min. Finally, samples were embedded in resin at 60°C. The hardened resin was then cut into 5 nm sections and transferred onto a grid. The samples were analyzed with a transmission electron microscope (Philips TEM 208 or FEI Tecnai G2 Spirit BioTwin).

### Confocal laser scanning microscopy

*P*. *aeruginosa* ATCC 33354 was cultured as described for electron microscopy and FLG2-4 concentration was adjusted to an identical molecule:CFU ratio. Bacteria were incubated for 30 min, 60 min, and 90 min with the protein, washed twice with PBS and resuspended in 2% formaldehyde/0.2% glutaraldehyde/PBS to a theoretical OD_600nm_ of 1. The cells were then fixed for 1 h at room temperature and subsequently stored overnight on glass slides at 4°C in a wet chamber. The next day, the samples were dried at room temperature, washed three times with PBS, and incubated for 5 min in 50 mM NH_4_Cl in PBS. Two washing steps in PBT (PBS/0.1% BSA+0.05% Tween 20) were followed by a 5 min incubation in 50 mM Tris/1 mM EDTA. For blocking, the samples were treated for 20 min with 0.1% BSA/0.2% glycine/Tris buffered saline. Incubation with goat-anti-FLG2-4 (0.5 mg/ml, 1:50 in PBT) was done overnight at 4°C. After three washing steps in PBT the samples were incubated 1 h with AlexaFluor 488-labeled chicken-anti-goat IgG (Molecular Probes) 1:200 and the DNA stain DRAQ 5 (1:1000) in PBT. The samples were mounted with Mowiol and analyzed with a confocal laser scanning microscope (Philips/FEI CM 10).

### Bacterial subfractionation

Mid-log-phase cultures of *P*. *aeruginosa* PAO1 grown in brain heart infusion (BHI) medium were resuspended in 10 mM sodium phospate buffer supplemented with 1% TSB and adjusted to OD_600nm_ 0.6. Bacteria were exposed to 0.8 μM FLG2-4 for 30 min at 37°C. Periplasmic fractions were collected as described [70]. Briefly, bacterial pellets were washed in 10 mM sodium phosphate buffer with 20% sucrose and 1 mM EDTA (pH 8.0), incubated with agitation for 10 min at room temperature, and centrifuged at 10000 × g for 10 min. Bacterial pellets were resuspended in ice-cold H_2_O and shaken on ice for 10 min. The suspension was centrifuged at 10000 × g for 10 min at 4°C. Supernatants containing the periplasmic fractions were saved. To isolate the cytosolic content, the bacteria were resuspended in H_2_O prior to heat inactivation and lysis at 95°C for 5 min. After centrifugation the supernatants were saved as cytosolic fractions. To separate the inner and outer membrane fractions, the remaining pellets were treated with 1% sarkosyl in 30 mM Tris (pH 8.0) [71]. After centrifugation the supernatants were saved as inner membrane fractions and the sarkosyl-insoluble pellet as outer membrane fractions and finally resuspended in SDS PAGE loading buffer. The fractions were separated by SDS PAGE using 12% acrylamid gels and analyzed by Western Blot as described [23]. Inner membrane fractions were verified with antibodies directed against XcpY [72].

### Membrane permeabilization assays

The Lysozyme Lysis Assay was conducted as described earlier [73], except that chloramphenicol (15μg/ml) was used instead of streptomycin and the decrease in optical density was monitored at 595 nm. For the SYTOX Green uptake, mid-log-phase BHI cultures of *P*. *aeruginosa* PAO1 were suspended in 10 mM sodium phosphate buffer, supplemented with 1% TSB and adjusted to OD_600nm_ 0.2. SYTOX Green was added at 1 μM final concentration and bacteria were exposed to 0.8 μM FLG2-4, 2 μM Polymyxin B, or kept untreated. DNA-associated fluorescence (Excitation 485 nm/Emission 535nm) was time-dependently quantified using a Berthold Twinkle microplate fluorometer.

### Electromobility shift assay

pUC18 plasmid DNA was isolated with the GeneJET Plasmid Miniprep Kit (Thermo Scientific). To linearize the plasmid, pUC18 was digested with *Hind*III and purified with the GeneJET Extraction Kit (Thermo Scientific). Approximately 120 ng of supercoiled or linearized plasmid and increasing concentrations of FLG2 peptide fragments (0.3 μM, 0.9 μM, 1.8μM) were incubated for 30 min at room temperature in 670 mM Tris-HCl, pH 8.8, 160 mM (NH_4_)_2_SO_4_, 25 mM MgCl_2_, 0.1% Tween20. Samples were loaded immediately onto a 0.8% agarose gel in 0.5 x TAE pH 8.0 buffer, supplemented with 0.05% ethidium bromide, and run at a field strength of 5.7 V/cm. After electrophoresis, bands were visualized under UV light.

### *In vivo* formaldehyde crosslinking

Logarithmic grown *P*. *aeruginosa* PAO1 were washed and concentrated to an OD_600nm_ of 2 in 10 mM sodium phosphate/1% TSB. The amount of FLG2-4 used was about 2 x 10^7^ molecules per CFU. Samples were incubated at room temperature for 30 min and subsequently treated with formaldehyde (1% final) for 30 min. Crosslinking was stopped by addition of glycine (125mM final) and bacteria were washed. Cosslinked DNA was immediately isolated with peqGold TriFast according to the manufacture’s protocol and dot-blot analyses were performed using the anti-FLG2-4 antibody.

### PCR inhibition experiments

The influence of different FLG2 C-terminal peptides on a PCR was analyzed using a conventional *Taq* S polymerase (Genaxxon). Human keratinocyte cDNA, corresponding to 10 ng total RNA, was used as template in a 20 μl PCR reaction, including 200 μM dNTPs, 200 nM each primer and 0.5 U polymerase for 30 cycles. Primer pairs for the housekeeping gene GAPDH (5'-CCAGCCGAGCCACATCGCTC-3' (forward) and 5'-ATGAGCCCCAGCCTTCTCCAT-3' (reverse)) with an annealing temperature of 60°C were used. FLG2-4 was used in three different concentrations, 0.78μM (250 ng/20 μl PCR reaction), 0.32 μM (100 ng/20 μl), and 0.16 μM (50 ng/20 μl), whereas both hBD-2 and BSA were only used at 250 ng/20μl (2.88 μM and 0.19 μM, respectively). PCR samples were subsequently loaded on a 1.2% agarose gel containing 0.5 μg/ml ethidium bromide and were run in a 1 x TAE buffer system. Bands were visualized under UV light.

### *In vivo* replication assay

*E*. *coli* bearing plasmid pBR322 was grown in supplemented M9 at 30°C to mid-log-phase. Pelleted cultures were resuspended in 10 mM sodium phospate buffer supplemented with 1% TSB, adjusted to OD_600nm_ 0.2, and supplemented with 170 μg/ml chloramphenicol. Aliquots were treated with 0.8 μM FLG2-4 or kept untreated. Incubation was continued and samples were taken after 0, 60, 120, 180, 240 min, heat inactivated at 95°C, and subsequently analyzed by PCR. 1μl was used as template in a 20 μl PCR reaction including 200 μM dNTPs, 200 nM each primer and 0.5 U Taq S polymerase (Genaxxon) for 18 cycles. The sequences of the used pBR322 primer pair for the *bla* gene is 5'-TTGCCGGGAAGCTAGAGTAA-3' (forward) and 5'-GCTATGTGGCGCGGTATTAT-3' (reverse) with an annealing temperature of 60°C. As control for stalled chromosomal DNA replication the 16S rRNA gene was amplified using 5'- AGAGTTTGATCMTGGCTCAG-3' (forward) and 5'-TACGGYTACCTTGTTACGACTT-3' (reverse) with an annealing temperature of 56°C. The PCR samples were subsequently loaded on a 1.2% agarose gel containing 0.5 μg/ml ethidium bromide and run in a 1 x TAE buffer system. Bands were visualized under UV light.

### Accession numbers

Filaggrin-2: NP_001014364 XP_371313; Filaggrin: NP_002007 XP_048104 XP_378901; Human beta-defensin-2: AAC69554; Dermcidin: ABQ53649

## Supporting Information

S1 Fig Localization of filaggrin-2 in human epidermis. Immunohistochemical analyses of FLG2 in human skin sections. Healthy skin sections from palmar sites were stained with anti-FLG2-4 antibody (left panel). Specificity of antibody was confirmed by blocking the antibody with the antigen (right panel).
(PDF)

S2 Fig Effect of FLG2-4 on P. aeruginosa. Electron microscopy images of P. aeruginosa treated with FLG2-4 for the indicated time periods (right panel). Left panel: untreated controls
(PDF)

S3 Fig Localization of filaggrin-2 during induced bleb formation. Confocal laser scanning microscopy of FLG2-4 treated *P*. *aeruginosa* ATCC33354, upper left panel: phase contrast, upper right panel: FLG2-4 immunostaining, lower left panel: merged, lower right panel: merged image of untreated bacteria.
(PDF)

S4 Fig Electrophoretic shift assays of linear plasmid DNA. Linearized plasmid DNA (~120 ng) was incubated using increasing concentrations of the FLG2-C-terminal fragment (FLG2-C-Term), FLG2-B14, and FLG2-B13. Used concentrations are indicated below the lanes.
(PDF)

S5 Fig Replication inhibition assay. A: Specificity of PCR inhibition. FLG2-4 mediated PCR inhibition was challenged by addition of FLG2-B13 and FLG2-B14 to the reaction mix. 250 ng of each protein was used. B: PCR inhibition assay. Equal amounts of the cationic antimicrobial peptide human β-defensin (hBD)-2 and bovine serum albumine (BSA) were tested for their PCR inhibiting properties and compared to FLG2-4. 250 ng of each protein was used. C: PCR rescue. PCR reactions were performed in absence (-) and presence of (+) 0.8 μM FLG2-4 using increasing unit concentrations of Taq-polymerase (U Pol). D: *In vivo* replication assay. The effect of 0.8 μM FLG2-4 on pBR322 replication in Chloramphenicol-treated *E*. *coli* was monitored over indicated time periods. 1μl from the bacterial suspension was then used as template in a PCR reaction amplifying the 16S rRNA gene as growth arrest control. Control: PCR reaction without addition of any of the tested proteins. NTC: no template control. M: molecular mass marker.
(PDF)

S1 Table Antimicrobial activity of FLG2-4 tested against various *Pseudomonas* strains, cystic fibrosis clinical isolates, and different Pseudomonads in the radial diffusion assay. The MEC is defined as the “minimal effective concentration” by the x-axis intersect in the graphical analyses of radial diffusion assays. The MECs stated represent the x-axis intersect of a regression calculated over all measured clearing zone units, the 95% confidence interval is indicated in parentheses; n = 3–12. Shaded gray: turbid clearing zones
(PDF)

S2 Table Antimicrobial activity of FLG2-4 tested against various bacterial strains in the radial diffusion assay. The results are displayed as the mean clearing zone units ± SD (in parentheses) at the highest used concentrations of FLG2-4 (63.0μM); n = 3–12.
(PDF)
