# Supplementary material for: Skin-Derived C-Terminal Filaggrin-2 Fragments Are Pseudomonas aeruginosa-Directed Antimicrobials Targeting Bacterial Replication
Source: PLoS Pathog. 2015 Sep 15;11(9):e1005159. doi: 10.1371/journal.ppat.1005159 (PMC4570713; doi:10.1371/journal.ppat.1005159)
Supplement: S1 Fig — Immunohistochemical analyses of FLG2 in human skin sections. Healthy skin sections from palmar sites were stained with anti-FLG2-4 antibody (left panel). Specificity of antibody was confirmed by blocking the antibody with the antigen (right panel). (PDF) [file ppat.1005159.s001.pdf]

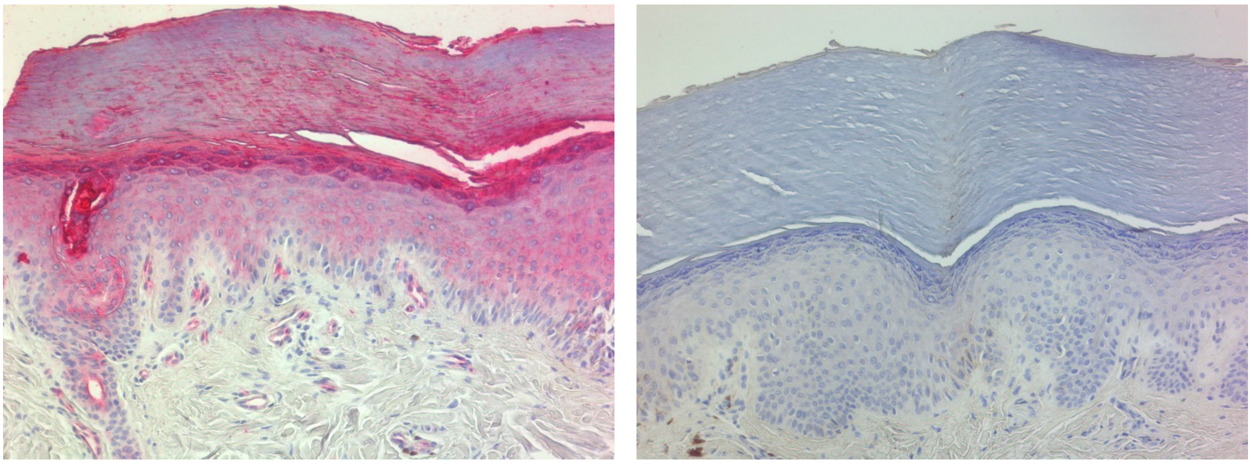

S1 Fig.: Localization of filaggrin-2 in human epidermis. Immunohistochemical analyses of FLG2 in human skin sections. Healthy skin sections from palmar sites were stained with anti-FLG2-4 antibody (left panel). Specificity of antibody was confirmed by blocking the antibody with the antigen (right panel).
