# Supplementary material for: Skin-Derived C-Terminal Filaggrin-2 Fragments Are Pseudomonas aeruginosa-Directed Antimicrobials Targeting Bacterial Replication
Source: PLoS Pathog. 2015 Sep 15;11(9):e1005159. doi: 10.1371/journal.ppat.1005159 (PMC4570713; doi:10.1371/journal.ppat.1005159)
Supplement: S2 Fig — Electron microscopy images of P. aeruginosa treated with FLG2-4 for the indicated time periods (right panel). Left panel: untreated controls (PDF) [file ppat.1005159.s002.pdf]

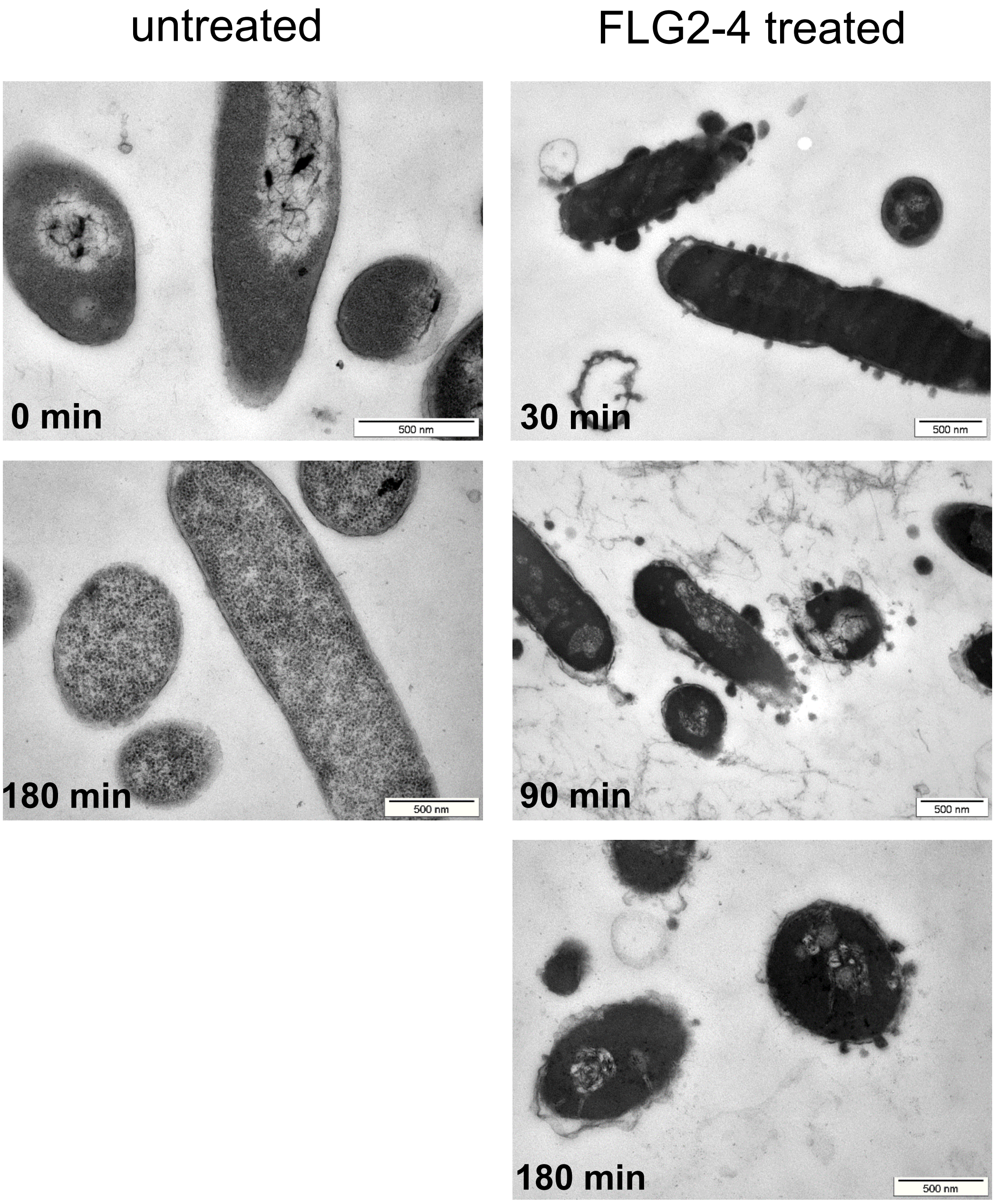

S2 Fig.: **Effect of FLG2-4 on *P. aeruginosa***. Electron microscopy images of *P. aeruginosa* treated with FLG2-4 for the indicated time periods (right panel). Left panel: untreated controls.
