# Supplementary material for: Skin-Derived C-Terminal Filaggrin-2 Fragments Are Pseudomonas aeruginosa-Directed Antimicrobials Targeting Bacterial Replication
Source: PLoS Pathog. 2015 Sep 15;11(9):e1005159. doi: 10.1371/journal.ppat.1005159 (PMC4570713; doi:10.1371/journal.ppat.1005159)
Supplement: S3 Fig — Confocal laser scanning microscopy of FLG2-4 treated P. aeruginosa ATCC33354, upper left panel: phase contrast, upper right panel: FLG2-4 immunostaining, lower left panel: merged, lower right panel: merged image of untreated bacteria. (PDF) [file ppat.1005159.s003.pdf]

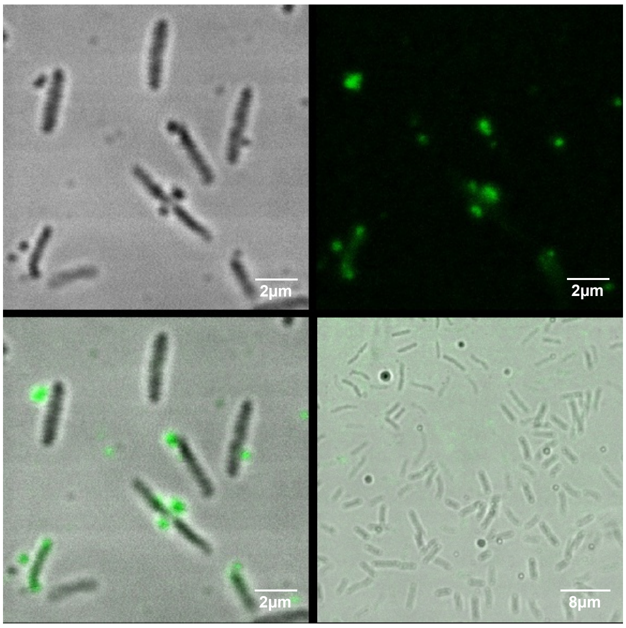

S3 Fig.: Localization of filaggrin-2 during induced bleb formation. Confocal laser scanning microscopy of FLG2-4 treated *P. aeruginosa* ATCC33354, upper left panel: phase contrast, upper right panel: FLG2-4 immunostaining, lower left panel: merged, lower right panel: merged image of untreated bacteria.
