# Supplementary material for: Skin-Derived C-Terminal Filaggrin-2 Fragments Are Pseudomonas aeruginosa-Directed Antimicrobials Targeting Bacterial Replication
Source: PLoS Pathog. 2015 Sep 15;11(9):e1005159. doi: 10.1371/journal.ppat.1005159 (PMC4570713; doi:10.1371/journal.ppat.1005159)
Supplement: S4 Fig — Linearized plasmid DNA (~120 ng) was incubated using increasing concentrations of the FLG2-C-terminal fragment (FLG2-C-Term), FLG2-B14, and FLG2-B13. Used concentrations are indicated below the lanes. (PDF) [file ppat.1005159.s004.pdf]

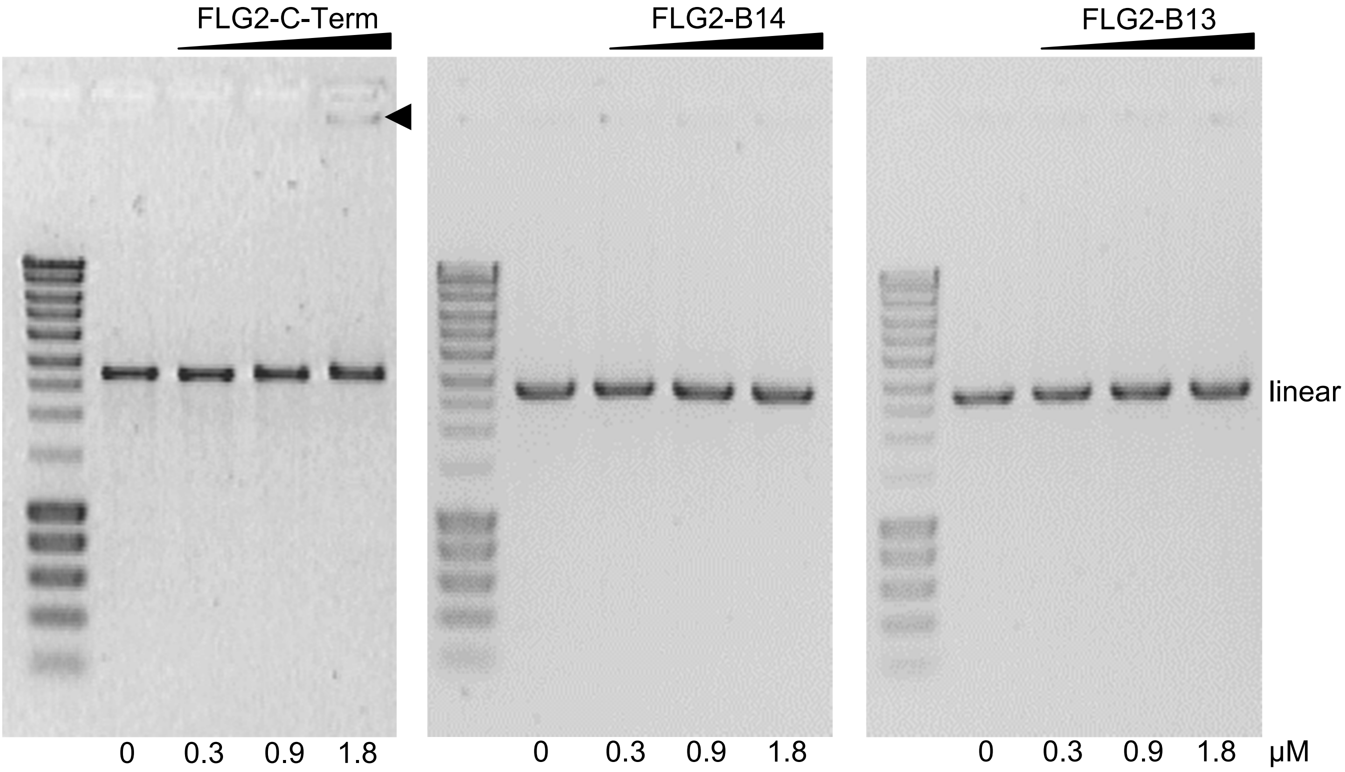

S4 Fig.: Electrophoretic shift assays of linear plasmid DNA. Linearized plasmid DNA (~120 ng) was incubated using increasing concentrations of the FLG2-C-terminal fragment (FLG2-C-Term), FLG2-B14, and FLG2-B13. Used concentrations are indicated below the lanes.
