# Supplementary material for: Skin-Derived C-Terminal Filaggrin-2 Fragments Are Pseudomonas aeruginosa-Directed Antimicrobials Targeting Bacterial Replication
Source: PLoS Pathog. 2015 Sep 15;11(9):e1005159. doi: 10.1371/journal.ppat.1005159 (PMC4570713; doi:10.1371/journal.ppat.1005159)
Supplement: S5 Fig — A: Specificity of PCR inhibition. FLG2-4 mediated PCR inhibition was challenged by addition of FLG2-B13 and FLG2-B14 to the reaction mix. 250 ng of each protein was used. B: PCR inhibition assay. Equal amounts of the cationic antimicrobial peptide human β-defensin (hBD)-2 and bovine serum albumine (BSA) were tested for their PCR inhibiting properties and compared to FLG2-4. 250 ng of each protein was used. C: PCR rescue. PCR reactions were performed in absence (-) and presence of (+) 0.8 μM FLG2-4 using increasing unit concentrations of Taq-polymerase (U Pol). D: In vivo replication assay. The effect of 0.8 μM FLG2-4 on pBR322 replication in Chloramphenicol-treated E. coli was monitored over indicated time periods. 1μl from the bacterial suspension was then used as template in a PCR reaction amplifying the 16S rRNA gene as growth arrest control. Control: PCR reaction without addition of any of the tested proteins. NTC: no template control. M: molecular mass marker. (PDF) [file ppat.1005159.s005.pdf]

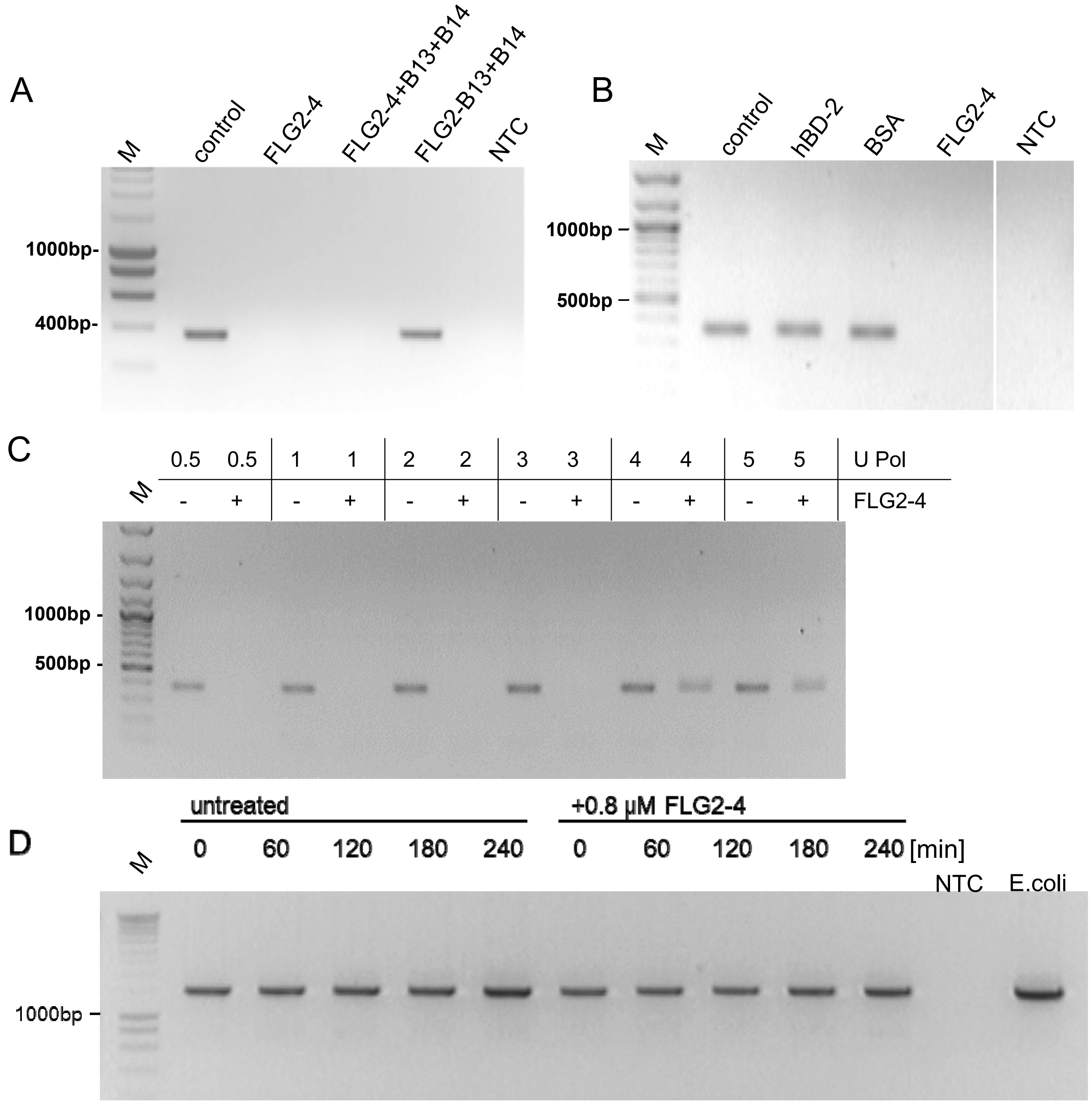

S5 Fig.: Replication inhibition assay. A: Specificity of PCR inhibition. FLG2-4 mediated PCR inhibition was challenged by addition of FLG2-B13 and FLG2-B14 to the reaction mix. 250 ng of each protein was used. B: PCR inhibtion assay. Equal amounts of the cationic antimicrobial peptide human β-defensin (hBD)-2 and bovine serum albumine (BSA) were tested for their PCR inhibiting properties and compared to FLG2-4. 250 ng of each protein was used. C: PCR rescue. PCR reactions were performed in absence (-) and presence of (+) 0.8 µM FLG2-4 using increasing unit concentrations of Taq-polymerase (U Pol).

D: *In vivo* replication assay. The effect of 0.8 µM FLG2-4 on pBR322 replication in Chloramphenicol-treated *E. coli* was monitored over indicated time periods. 1µl from the bacterial suspension was then used as template in a PCR reaction amplifying the 16S rRNA gene as growth arrest control. Control: PCR reaction without addition of any of the tested proteins. NTC: no template control. M: molecular mass marker.
