# Supplementary material for: Skin-Derived C-Terminal Filaggrin-2 Fragments Are Pseudomonas aeruginosa-Directed Antimicrobials Targeting Bacterial Replication
Source: PLoS Pathog. 2015 Sep 15;11(9):e1005159. doi: 10.1371/journal.ppat.1005159 (PMC4570713; doi:10.1371/journal.ppat.1005159)
Supplement: S1 Table — The MEC is defined as the “minimal effective concentration” by the x-axis intersect in the graphical analyses of radial diffusion assays. The MECs stated represent the x-axis intersect of a regression calculated over all measured clearing zone units, the 95% confidence interval is indicated in parentheses; n = 3–12. Shaded gray: turbid clearing zones (PDF) [file ppat.1005159.s006.pdf]

S1 Table: Antimicrobial activity of FLG2-4 tested against various *Pseudomonas* strains, cystic fibrosis clinical isolates, and different Pseudomonads in the radial diffusion assay. The MEC is defined as the “minimal effective concentration” by the x-axis intersept in the graphical analyses of radial diffusion assays. The MECs stated represent the x-axis intersept of a regression calculated over all measured clearing zone units, the 95% confidence interval is indicated in parentheses; n=3-12. Shaded gray: turbid clearing zones.

radial diffusion assay

**other *Pseudomonas***	**MEC [µM]**	**clinical *Pseudo-monas* isolates**	**MEC [µM]**	**other Pseudomonads**	**MEC [µM]**
***P. aeruginosa* ATCC 10145**	**0.7 (0.2-1.5)**	**CF 636**	**0.9 (0.1-2.3)**	***P. stutzeri* RV A2/1990**	**0.3 (0.2-0.5)**
***P. aeruginosa* ATCC 33348**	**1.0 (0.8-1.2)**	**CF 640**	**0.1 (0.02-0.4)**	***P. syringae* ATCC 10205**	**0.9 (0.3-1.8)**
***P. aeruginosa* ATCC 33358**	**1.1 (0.1-2.9)**	**CF 645**	**0.3 (0.1-0.5)**	***P. paucimobilis* RV A2/1994**	**1.9 (1.2-2.6)**
***P. aeruginosa* ATCC 39324**	**0.1 (0.05-0.3)**	**CF 646**	**0.4 (0.02-1.2)**	***P. fluorescens* ATCC 49323**	**0.2 (0.001-1.2)**
***P. aeruginosa* PAO1**	**0.7 (0.2-1.5)**			***P. putida* RV A1/2000**	**7.0 (1.0-12.7)**
