# Supplementary material for: Skin-Derived C-Terminal Filaggrin-2 Fragments Are Pseudomonas aeruginosa-Directed Antimicrobials Targeting Bacterial Replication
Source: PLoS Pathog. 2015 Sep 15;11(9):e1005159. doi: 10.1371/journal.ppat.1005159 (PMC4570713; doi:10.1371/journal.ppat.1005159)
Supplement: S2 Table — The results are displayed as the mean clearing zone units ± SD (in parentheses) at the highest used concentrations of FLG2-4 (63.0μM); n = 3–12. (PDF) [file ppat.1005159.s007.pdf]

S2 Table: Antimicrobial activity of FLG2-4 tested against various bacterial strains in the radial diffusion assay. The results are displayed as the mean clearing zone units ± SD (in parentheses) at the hightest used concentrations of FLG2-4 (63.0µM); n=3-12.

radial diffusion assay of FLG2-4

strain	clearing zone units	strain	clearing zone units	strain	clearing zone units	strain	clearing zone units
E. coli **ATCC 11775**	32.9 (± 3.3)	***P. aeruginosa*** **ATCC 33358**	15.8 (± 4.9)	**CF 640**	31.7 (± 2.9)	***P. syringae*** **ATCC 10205**	37.0 (± 9.7)
P. aeruginosa **ATCC 33354**	39.6 (± 4.5)	***P. aeruginosa*** **ATCC 39324**	25.8 (± 6.6)	**CF 645**	53.3 (± 2.9)	***P. paucimobilis*** **RV A2/1994**	45.8 (± 2.0)
P. aeruginosa **ATCC 10145**	27.5 (± 2.7)	***P. aeruginosa*** **PAO1**	32.5 (± 6.5)	**CF 646**	26.3 (± 4.8)	***P. fluorescens*** **ATCC 49323**	22.5 (± 5.0)
P. aeruginosa **ATCC 33348**	35.0 (± 2.5)	**CF 636**	28.3 (± 2.9)	***P. stutzeri*** **RV A2/1990**	61.7 (± 9.7)	***P. putida*** **RV A1/2000**	21.3 (± 5.8)
